# Gas-Diffusion Microextraction (GDME) Combined with Derivatization for Assessing Beer Staling Aldehydes: Validation and Application

**DOI:** 10.3390/foods10081704

**Published:** 2021-07-22

**Authors:** Inês M. Ferreira, Daniel O. Carvalho, Marco Gomes da Silva, Luís Ferreira Guido

**Affiliations:** 1 REQUIMTE/LAQV, Departamento de Química e Bioquímica, Faculdade de Ciências, Universidade do Porto, Rua do Campo Alegre 687, 4169-007 Porto, Portugal; ines.filipa.mourao.ferreira@gmail.com (I.M.F.); daniel.carvalho@fc.up.pt (D.O.C.); 2 REQUIMTE/LAQV, Departamento de Química, Faculdade de Ciências e Tecnologia, Universidade Nova de Lisboa, 2829-516 Caparica, Portugal; mdr@fct.unl.pt

**Keywords:** beer aging, carbonyl compounds, Strecker aldehydes, GDME, microextraction

## Abstract

In this work, a gas-diffusion microextraction (GDME) methodology was optimized and validated for the analysis of selected staling aldehydes (furfural (FURF), 2-methylpropanal (2-MP), 2-methylbutanal (2-MB), 3-methylbutanal (3-MB), and acetaldehyde (ACET)) during natural and forced aging of beer. The methodology was optimized considering time, temperature of extraction, and derivatizing agent. Using 4-hydrazinobenzoic acid (HBA) as a derivatizing agent, the performance of the method was evaluated by assessing several parameters such as detection limits (ranging from 1.2 to 1857.7 µg/L for 2-MB and ACET, respectively), quantification limits (ranging from 3.9 to 6192.4 µg/L for 2-MB and ACET, respectively), recoveries (higher than 96%), intraday and interday precisions (lower than 3.4 and 9.2%, respectively), and linearity (r^2^ ≥ 0.995). During beer aging, higher content of Strecker aldehydes and FURF were found, while no significant variations in ACET levels were observed. In general, the aldehydes content assessed for beers stored at 37 ± 1 °C for 7 and 14 days mimics that observed for beers stored at 20 ± 2 °C for 3 and 6 months, respectively. Lower temperatures of storage (4 ± 1 °C) delayed the development of staling aldehydes. Based on PCA analysis, the content of staling aldehydes and beer color were responsible for 91.39% of the variance among the analyzed samples, and it was demonstrated that these are key parameters to discriminate fresh from aged beers. The results herein presented showed that the proposed analytic methodology is a valuable strategy for the characterization and quantification of important staling aldehydes in beer with a potential application in the quality control of beer during storage.

## 1. Introduction

Beer is a complex matrix of more than 3000 different compounds in an aqueous environment. Thereby, it is not surprising that the organoleptic quality of beer changes over time, and consequently, the majority of beer companies are interested and dedicated to perceiving these alterations in order to preserve beer “freshness” [1]. The occurrence of beer aging or staling has been intensively investigated by the brewing industry to understand and control it, but the involved mechanisms are still not fully understood [2,3]. Beer’s flavor is not a static phenomenon and is one of the greatest challenges in the brewing industry and the most important criterion for evaluation of beer quality [4,5]. During beer storage, a constant decrease in bitterness together with an increase of sweet and toffee-like aromas and flavors are perceived. In addition, a prompt formation of ribes flavor has been described during beer aging, associated with a characteristic odor of blackcurrant leaves and later by a cardboard flavor, directly linked to beer staling [6,7]. The compounds that mainly contribute to beer staling are aldehydes [8,9,10], whose concentration increase during beer distribution and storage, leading to the appearance of aged flavors such as nut, fruit, cardboard, caramel, and bready, among others [11]. The most important staling aldehydes identified in beer comprise fatty acid oxidation aldehydes (e.g., hexanal, *E*-2-nonenal), Maillard reaction products (e.g., furfural (FURF) and 5-hydroxymethylfurfural (5-HMF)), and Strecker degradation products (e.g., 2-methylpropanal (2-MP), 2-methylbutanal (2-MB), 3-methylbutanal (3-MB), phenylacetaldehyde, methional) [12]. Maillard reaction products (MRP) are formed during heat processing at pH values of around 4–7, contributing to the beer’s flavor and changes in color as a result of nonenzymatic browning and caramelization [6,13]. In general, 5-HMF and FURF are quantitatively the most important Maillard products in beer, being associated to caramellic and bready flavors [6]. Further important flavoring compounds in beer are Strecker aldehydes, which may be generated from the reaction of amino acids with α-dicarbonyl compounds via Strecker degradation or through oxidation of higher alcohols [14]. The presence of their precursors in beer leads to the formation of several Strecker aldehydes, such as 2-MP, derived from valine; 3-MB, derived from leucine; and 2-MB, derived from isoleucine. In beer, 2-MP, 2-MB, and 3-MB are considered to be responsible for the “malty” character [14]. ACET is the predominant aldehyde in beer, representing approximately 60% of the total aldehydes content [15], and, when existing above its flavor threshold (approximately 25 mg/L), it imparts an undesirable cut grass or green apple character [16]. The analysis of flavor compounds in beer has been continuously optimized to achieve better sensitivity and specificity. In the last 10 years, different extraction techniques have been applied for the analysis of carbonyl compounds in alcoholic beverages, such as headspace solid-phase microextraction (HS-SPME) [17,18] and stir bar sorptive extraction (SBSE) [19,20]. Derivatization techniques are also a good strategy to improve the volatility and extraction of semi-volatile compounds, as well as to improve its stability [21] and detectability during LC-UV analysis [22]. Our research group has applied the gas-diffusion microextraction (GDME) extraction procedure for the identification of several carbonyl compounds in different liquid and solid matrices ([23,24,25,26]). This technique consists of the extraction of volatile analytes through a permeable membrane to an acceptor solution, combined with a derivatization step (acceptor solution containing the derivatizing agent). GDME exhibits many advantages since it is a faster, simpler, and cheaper alternative method for the determination of aldehydes, comparing to commonly applied methods. In addition, GDME allows both isolation, concentration and derivatization of the analytes in one simple and fast step. Bearing this in mind, the present work aimed the validation of a methodology based on simultaneous GDME extraction and derivatization, for the identification and quantification of staling aldehydes in beer. The validated procedure was applied to investigate the impact of storage time and temperature on the concentration of several aldehydes (ACET, FURF, 2-MP, 2-MB, and 3-MB).

## 2. Materials and Methods

### 2.1. Chemicals and Solutions

All reagents were of analytical grade. Ultrapure water was obtained from a Direct-Q 3UV water purification system (Millipore Iberian, Spain) and used for all aqueous solution preparation and glassware washing. HPLC gradient grade acetonitrile (>99.9%) was purchased from Fisher Scientific (Waltham, MA, USA). Formic acid (90–100%, a.r.) and ethanol (96%, p.a.) were purchased from Chem Lab (Zedelgem, Belgium). Hydrochloric acid (37%) was obtained from Merk (Darmstadt, Germany). Acetic acid glacial (Reag. Ph. Eur., PA-ACS-ISO) was purchased from Panreac (Barcelona, Spain). 4-hydrazinobenzoic acid (HBA, 97%), 2,4-dinitrophenylhydrazine (DNPH, 97%), ammonium acetate (>98%, reagent grade), acetaldehyde (99%), 2-furaldehyde (furfural, 99%), isobutyraldehyde (2-methylpropanal, 99%), isovaleraldehyde (3-methylbutanal, 95%), 2-mehtylbutyraldehyde (2-methylbutanal, 95%), 4-fluorobenzaldehyde (98%), were purchased from Sigma-Aldrich (St. Louis, MO, USA).

The DNPH solution (0.3%, *w*/*v*) was weekly prepared in acetonitrile/40 mM HCl (1:1). The HBA stock solution (2500 mg/L) was weekly prepared in 0.1 mol/L HCl and protected from the light. The HBA working solution (300 mg/L) was daily prepared in acetonitrile/0.1 mol/L (1:1) from the stock solution. Aldehydes stock solutions (2.0 g/L for 2-MB, 3-MB and 2-MP, 100 g/L for FURF, 65.0 g/L for ACET and 120.0 g/L for the internal standard (IS, 4-fluorobenzaldehyde) were prepared in acetonitrile and maintained at −20 °C. Working solutions were prepared by diluting stock solutions in acetonitrile.

### 2.2. Sampling and Aging Assays

All beer samples were kindly provided by Super Bock Group (Porto, Portugal). Beers were naturally and forcibly aged. During forced aging, beers were stored in an oven (Raypa, Incuterm, Barcelona, Spain) under controlled temperature at 37 ± 1 °C, in order to reproduce warm storage conditions. The carbonyl compounds levels were monitored at 7, 14, 21, 35, 49, 63, and 90 days. Additionally, the study was also conducted in natural aging conditions. For this purpose, beers were stored for 3 and 6 months at room temperature (20 ± 2 °C) and at 4 ± 1 °C (considered as control samples).

### 2.3. Experimental Procedure

#### 2.3.1. Extraction Apparatus and Operation Mode

The basic extraction principles of GDME were previously described by Pacheco and co-workers [24]. To perform the extraction of the volatile compounds with the headspace module: (1) 50 mL of sample (model solution 5% ethanol or beer) was placed on a glass flask (100 mL) to which 50 µL of IS (12.5 mg/L) was added; (2) the flask was tightly closed with the respective cap containing the GDME extraction device with the PTFE hydrophobic membrane (Mitex Membrane Filters, Milipore, Darmstadt, Germany); (3) the flask containing the sample was placed into a water bath, with continuous agitation; (4) 0.5 mL of derivatizing agent (DNPH or HBA) was added to the GDME extraction device.

The extraction of carbonyl compounds with the immersed module was performed as described: (1) 25 mL of sample (model solution 5% ethanol or beer) was placed on the thermostatized chamber (Metrohm vessel with thermostat jacket, 30 mL), with continuous agitation, to which 50 µL of IS (12.5 mg/L) was added; (2) the extractor device with the PTFE membrane was attached to the lid of the chamber to create a closed environment; (3) 0.5 mL of derivatizing agent (HBA) was added to the extractor device. At the end of the defined extraction period, the extract was collected and transferred into a vial to be analyzed by HPLC-DAD.

#### 2.3.2. Extraction Conditions Study

In this work, several parameters influencing the extraction of beer carbonyl compounds were evaluated: time and temperature of extraction, the derivatizing agent (DNPH and HBA), and two different extraction approaches (headspace or immersed module). The optimization of the extraction parameters was performed using aldehydes standards (1.0 mg/L) prepared in model solutions (5% ethanol). The experiments started with the study of the impact of time (5, 10, 20, and 30 min) and temperature (40, 50, and 60 °C) on extraction for both DNPH and HBA.

In a first approach, the optimal temperature and time of extraction using both derivatizing agents were evaluated with the headspace module. For the study of time, the temperature was fixed at 40 °C. Then, the influence of temperature was studied for the optimized extraction time for both derivatizing agents. Once the optimal conditions were defined (30 min and 40 °C for DNPH and 20 min and 40 °C for HBA), the main differences between DNPH and HBA were evaluated to select the best derivatizing agent. Then, the extraction efficiency was evaluated in the headspace and immersed module conditions by using the previously optimized conditions.

#### 2.3.3. Optimized Extraction Conditions

50 mL of beer were placed in the glass flask (100 mL) and 50 µL of IS were added (in a final concentration of 12.5 mg/L); 0.5 mL of the derivatizing solution (300 mg/L HBA) was placed inside the GDME device which is in the headspace of the glass flask. The extraction was performed according to the optimized conditions: 20 min at 40 °C. The GDME module was washed using ultrapure water between extractions, and the membrane was changed every day.

### 2.4. HPLC-DAD Analysis

The chromatographic analyses were performed using a Scientific Dionex Ultimate 3000 HPLC system equipped with a quaternary pump (LPG-3400SD), an autosampler (ACC-3000), a column oven, and a diode-array detector (DAD-3000) from Thermo Scientific (Waltham, MA, USA). The stationary phase was a C18 Gemini NX column (150 × 4.6 mm; 3 µm) equipped with a guard column (Gemini C18, 4.0 × 3.0 mm) from Phenomenex (Torrence, CA, USA). The column was maintained at 30 °C during the chromatographic separations.

The analysis of DNPH-derivatives was based on a chromatographic method described by Cordeiro and co-workers [26], with slight modifications. The elution of DNPH-derivatives was performed in a gradient mode using acetonitrile (A) and acetate buffer (10 mM, pH 4.8) (B) as follows: 0–20 min: 50% to 65% A; 20–45 min: 65% to 100% A; 45–50 min: 50% A; and 50–55 min: 50% A. The flow rate was set at 0.45 mL·min^−1^, the sample volume injected was 20 µL, and the detection was performed at 360 nm.

The analysis of HBA-derivatives by HPLC-DAD was performed as described by Lima and co-workers [23], with slight modifications. The elution of HBA-derivatives was performed in a gradient mode using acetonitrile (A) and formic acid, 0.1% (B), using the following conditions: 0–25 min: 27% to 43% A; 25–32 min: 43% to 51% A; 32–40 min: 51% to 27% A, and 40–45 min: 27% A. The flow rate was set at 0.45 mL·min^−1^. The injection volume was 20 µL and the detection wavelength was set at 320 nm. Data acquisition and analysis were performed using Chromaleon software (version 7, Thermo Scientific, Waltham, MA, USA).

For quantification purposes, the internal standard calibration method was used. To all the calibration solutions, a fixed concentration of IS (12.5 mg/L) was added. The analytical signal was calculated as the ratio of the analyte signal to the IS signal for all the calibration solutions, and the linear regression of relative signal vs. relative concentration was assessed for each compound at the established concentration ranges.

### 2.5. Beer Color Determination

Beer color was measured according to the EBC Method 9.6—Color of Beer: spectrophotometric method [27]. Beer samples were degassed by gently stirring with a magnetic stirrer at low speed before the measurements using a UV-VIS-NIR Scanning Spectrophotometer, UV-3101PC (Shimadzu, Kyoto, Japan). The color in EBC units (European Brewing Convention) was calculated as shown in Equation (1).

(1) $$Color\;\left(EBC\;Units\right)=A\times f\times 25$$

where *A* is the absorbance at 430 nm and *f* is the dilution factor.

### 2.6. Statistical Analysis

All the experiments were conducted in triplicate and results are expressed as mean ± standard deviation (SD). The significant differences between the values of aldehydes concentration in fresh and aged beers were evaluated by the analysis of variance (ANOVA) followed by Tukey post-hoc test. The significant differences between the slopes of the established calibration curves (in model systems and in beer) were assessed applying the *t*-test. Statistically significant differences were considered for *p* < 0.05, considering a 95% confidence level. These statistical analyses were performed using IBM Statistics 27 (New York, NY, USA). The principal component analysis (PCA) was applied to the data set of all analyses performed for fresh and aged (naturally and forcing) beers to reduce the number of variables (aldehydes concentration) and to detect a pattern in the relationship between the variables and the beer samples. PCA plots were performed using XLSTAT software (v.2021.2.1.1129, Addinsoft, New York, NY, USA).

## 3. Results and Discussion

### 3.1. Study of Temperature and Time of Extraction for DNPH and HBA

Different parameters such as temperature and time of extraction can affect the GDME extraction’s efficiency [24,25,28]. Thus, the optimal conditions to analyze beer staling aldehydes using GDME were evaluated by testing two derivatizing agents (DNPH and HBA). The study was conducted in model solutions (5%, *v*/*v*), and blank experiments were carried out to evaluate the possible conversion of ethanol into acetaldehyde by the application of temperature (40 °C) during the extraction procedure. A conversion of between 4% and 5% was found, which does not represent a significant impact on the studied levels of acetaldehyde. DNPH is one of the most used derivatizing agents for beer analysis. The DNPH-derivatives (hydrazones) have a higher absorption coefficient and are improved hydrophobic retentions, and they can be easily separated by HPLC and detected by UV spectroscopy at 360 nm [28]. HBA reacts with aldehydes and leads to the formation of stable imines. It was also successfully applied for the analysis of low molecular aldehydes in complex alcoholic beverages in combination with HPLC-UV (at 320 nm) [23].

**Figure 1:**
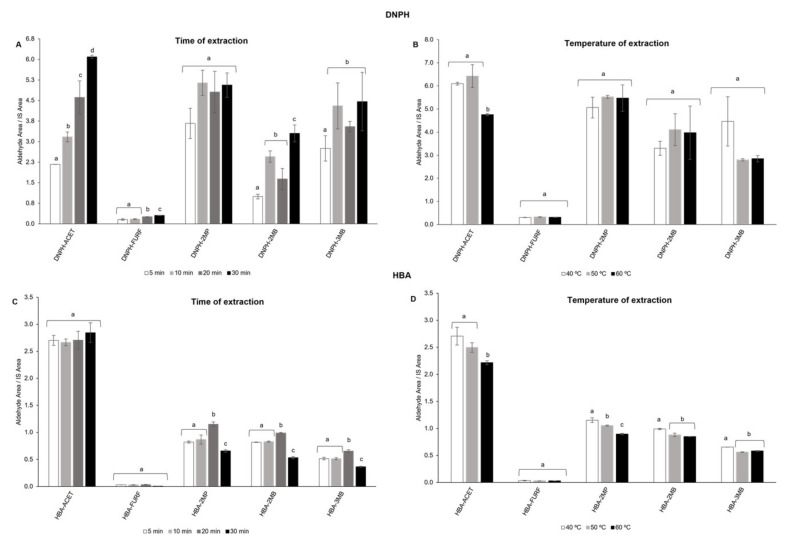
Optimization studies for headspace extraction of a mixture of aldehydes (1 mg/L) in model solution (5% ethanol), using DNPH and HBA as derivatizing agents: (**A**) effect of time (40 °C) and (**B**) temperature (30 min), on DNPH-derivatives extraction; (**C**) effect of time (40 °C) and (**D**) temperature (30 min), on HBA-derivatives extraction. Bars represent mean ± SD (*n* = 3). The different superscript letters represent significant statistical differences (*p* < 0.05), for each compound, at different times and temperatures.

In a first approach, GDME in headspace was applied using DNPH as a derivatizing agent, to evaluate the optimal time and temperature of extraction (from 5 to 30 min and 40 to 60 °C, respectively). At first, the time of extraction was evaluated by setting the temperature at 40 °C (Figure 1A).

According to the obtained results, the maximum signals for the tested aldehydes were achieved after 30 min of extraction. The effect of temperature on extraction efficiency was further evaluated for the selected extraction time of 30 min (Figure 1B). The three tested temperatures (40, 50 and 60 °C) did not affect the performance of extraction since no statistical differences were obtained for the analytical signals, except for ACET, which showed lower signals at 60 °C. With a boiling point of 20.8 °C, acetaldehyde is among the most highly volatile carbonyls. According to these results, 30 min and 40 °C were the chosen conditions for the extraction using DNPH as a derivatizing agent.

The same parameters (time and temperature) were further evaluated, using HBA as a derivatizing agent. The time of extraction was studied by setting the temperature at 40 °C (Figure 1C). After 20 min, it was observed a statistically significant increase (*p* < 0.05) of the analytical signal for Strecker aldehydes (approximately 1.3-fold for 2-MP, 2-MB, and 3-MB, compared to 10 min extraction). In contrast, for FURF and ACET, no statistically significant differences were observed. Hence, the effect of temperature on extraction efficiency was evaluated during 20 min of extraction (Figure 1D). Using HBA as a derivatizing agent, the extraction at 40 °C allowed a higher extraction efficiency for Strecker aldehydes, while no differences were found for FURF at the tested temperatures. In addition, no significant differences were obtained for ACET at 40 and 50 °C, while a lower signal was observed at 60 °C. Considering these results, 20 min and 40 °C were selected for the GDME extraction using HBA as a derivatizing agent.

### 3.2. Derivatizing Agent Choice (DNPH vs. HBA)

The potential use of DNPH and HBA for the analysis of beer staling aldehydes were investigated. Both derivatizing agents were applied for the detection of carbonyl compounds in several matrices, namely in alcoholic beverages [23,25]. This study intended to evaluate differences in selectivity, sensitivity, and efficiency among these derivatizing agents.

GDME in headspace was applied with a standard solution of aldehydes (1 mg/L), in a model solution, using both DNPH and HBA. The results, comparing the two derivatizing agents, are shown in Table 1.

DNPH exhibited higher levels of contamination, which hinders the proper identification of the analytes of interest. Moreover, HBA allowed better sensitivity for IS (approximately 70% higher comparing to DNPH). However, in spite of a slight decrease in the signal intensity (*p* < 0.05) for the majority of the studied compounds, lower variability was achieved for HBA comparing to DNPH. According to these results, HBA was chosen for further studies allowing better repeatability and acceptable levels of sensitivity.

### 3.3. Headspace Extraction vs. Immersed Extraction

After the selection of HBA as a derivatizing agent, the following studies aimed to evaluate the influence of the use of headspace or immersed module. Comparing both extraction methods, higher peak areas were achieved for FURF with the GDME module immersed in the sample. In contrast, when headspace extraction was used, higher peak areas were found for ACET, 2-MP, 3-MB, 2-MB and IS (Table 2).

The immersed module extraction was revealed to be more advantageous for the identification of FURF due to better extractability (1.5-fold higher comparing to headspace module extraction). However, when immersed extraction was applied, higher RSD values were obtained for intraday and interday precisions (ranging from 4.6% (2-MB) to 10.3% (FURF) and from 13.4% (IS) to 26.3% (3-MB), respectively). In contrast, lower RSD values were observed for intraday and interday precisions when headspace extraction was applied (ranging from 2.7% (2-MP) to 8.1% (IS) and from 6.9% (2-MP) to 9.7% (ACET), respectively). Intraday and interday precisions were determined by performing the assay of ten fresh spiked beers in the concentration range shown in Table 2. The differences between RSDs obtained, comparing headspace and immersed extractions, can be explained by the large amounts of CO_2_ and foam observed in the tested beers. The foam creates a natural barrier between the donor and the acceptor solution, hindering the analytes’ diffusion through the membrane to the acceptor solution when immersed extraction was used. Taking into consideration the previously mentioned, headspace extraction was selected.

### 3.4. Analytical Features of the Methodology

To evaluate the performance of the developed method, matrix effect, linearity, detection, and quantification limits (LOD and LOQ), recovery and intra- and interday precisions were determined using the optimized experimental conditions: 20 min extraction in headspace at 40 °C and using HBA as a derivatizing agent. The results are summarized in Table 3. The addition of 4-fluorobenzaldehyde as IS was important to balance the possible loss of volatile carbonyls during the sample extraction procedure.

Linearity and the influence of potential matrix effects were evaluated by comparing the slopes of calibration curves prepared both in model solutions (5% ethanol) and in fresh beers after GDME extraction. The study was conducted for the linear ranges of aldehydes described in Table 3, based on the expected content in fresh and aged beers. The comparison of the slopes of the calibration curves prepared in model solution and in spiked samples showed that the observed differences were not statistically significant (*p* > 0.05), which indicated the absence of matrix effect in the analysis. For this reason, and for quantification purpose, an internal standard calibration method was used. Calibration curves showed adequate linearity for the selected concentrations range (Table 3), with determination coefficients (r^2^) ranging from 0.995 to 0.999 for ACET and the other aldehydes, respectively. Detection and quantification limits (LOD and LOQ) were calculated, for all the compounds, as three- and ten-times standard deviation of the regression divided by the slope, respectively. The determined LODs range between 1.2 and 1857.7 µg/L for 2-MB and ACET, respectively. The determined LOQs range between 3.9 and 6192.4 µg/L for 2-MB and ACET, respectively.

The intraday precision was evaluated by performing ten HBA-derivatives replicates of a spiked fresh beer sample on the same day at two levels of concentration (Table 3), considering the determined LOQ values as well as the minimal and maximal concentrations in fresh and aged beer samples. The interday precision was evaluated by analyzing five HBA-derivatives replicates of a spiked fresh beer sample (at the same concentration levels) on three different days. The intraday precision values varied between 1.0% and 3.4% (for 2-MP at high and low levels of concentration, respectively), while the interday precision values varied between 3.3% and 9.2% (for 3-MB at a high level of concentration and 2-MP at a low level of concentration).

Recovery studies were performed in triplicate by spiking fresh beer samples at two different concentration levels (Table 3), corresponding to approximately 2-fold (low level) and 6-fold (high level) the determined LOQ of each analyzed compound. The recovery values obtained are exhibited in Table 3 and ranged from 96% to 114% for 2-MB and 2-MP, respectively, for both low and high concentrations levels tested.

These results indicated that the novel analytical method herein presented is highly precise and accurate (intraday and interday precision RSD lower than 10% and recoveries from 96% to 114%) and therefore suitable for the analysis of staling aldehydes in beer matrices.

### 3.5. Influence of Natural and Forced Aging in Aldehyde Levels

**Figure 2:**
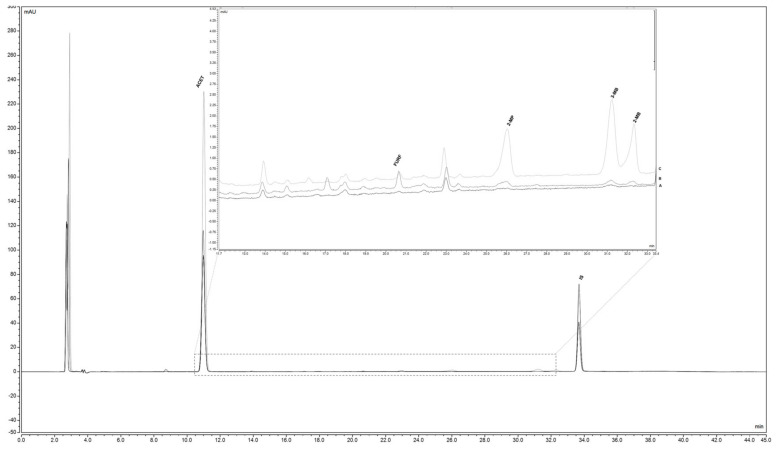
Chromatograms obtained at 320 nm in the study of (**A**) fresh beer sample; (**B**) beer maintained at 37 ± 1 °C for 63 days (forced aged beer) and (**C**) fresh beer sample spiked with 100 µg/L of 2-MP, 130 µg/L of 3-MB, and 2-MB, 150 µg/L of FURF, 8000 µg/L of ACET and 12,411 µg/L of IS.

The validated GDME method, using HBA as a derivatizing agent, was applied for the analysis of staling aldehydes (ACET, FURF, 2-MP, 2-MB, and 3-MB) in fresh, naturally, and forced aged beers. Typical chromatograms representing the aldehydes profile of fresh and forced aged beers (63 days at 37 ± 1 °C) are shown in Figure 2 (A and B, respectively). Additionally, to provide a clear identification of the studied aldehydes, a chromatogram of a spiked fresh beer is also presented (Figure 2C).

**Figure 3:**
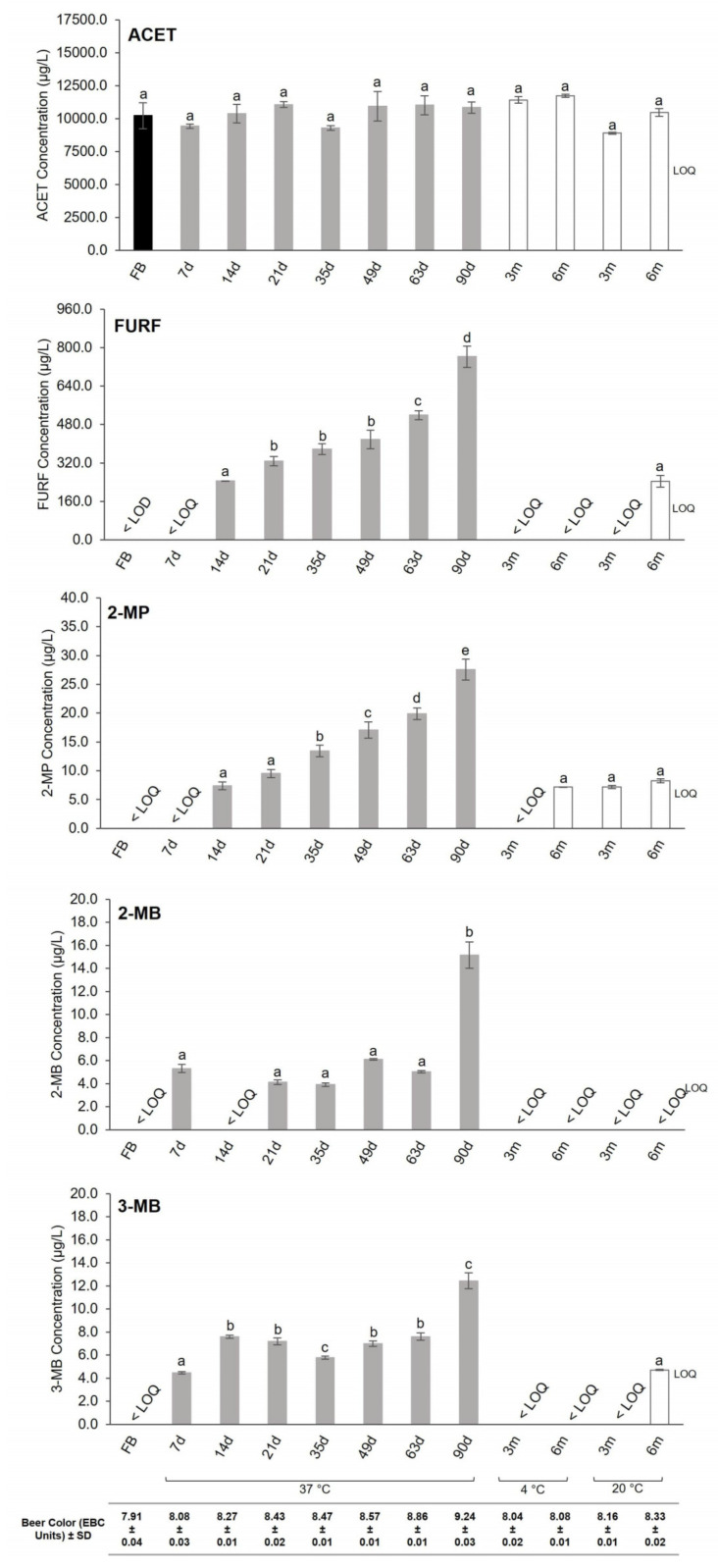
Concentration of the studied aldehydes and beer color (EBC Units) for fresh (FB), 3 months (3 m) and 6 months (6 m) naturally aged at 20 ± 2 °C and at 4 ± 1 °C, and forced aged beers (7–90 days at 37 ± 1 °C (7 d–90 d)). Bars represent mean ± SD (*n* = 3), and the different superscript letters represent differences considered statistically significant (*p* < 0.05) between the aging times for each individual compound.

The content of aldehydes in fresh and aged beers is reported in Figure 3. As observed, fresh beers showed a relatively low content of aldehydes comparing to aged beers. The major differences were found in forced aged beers considering FURF, 2-MP, 2-MB, and 3-MB contents. On the other hand, no statistically significant differences in ACET were observed during aging (Figure 3). Similar results were obtained by Vanderhaegen and co-workers, where the levels of ACET in beer remained constant during one year of natural aging [29]. The levels of ACET determined in fresh and aged beers are below the flavor threshold value (25 mg/L) [6], which means that no significant changes in the organoleptic characteristics and sensory quality of aged beers are expected.

No FURF was detected in fresh beers and low levels (below 154.5 µg/L) were observed in forced aged beers after 7 days. With extended storage times at 37 ± 1 °C, the concentration of FURF increased, attaining a maximum after 90 days (approximately 800 µg/L). This denoted an increase of about 200% (*p* < 0.05) comparing to beers stored for 14 days at 37 ± 1 °C. Previous works have reported levels of FURF between 287 and 457 µg/L in beers stored for 21 days at 40 °C [8,30], which are in accordance with the FURF contents of 327 ± 20 µg/L determined in forced aged beers for 21 days (Figure 3). Beers submitted to natural aging for 6 months showed higher levels of FURF (244 ± 24 µg/L) compared to fresh beers, but approximately 70% lower comparing to forced aged beers for 90 days. However, similar FURF contents (*p* > 0.05) were found for beers stored at 20 ± 2 °C for 6 months and 37 ± 1 °C for 14 days. These results are in accordance with the findings of Saison et al., who found FURF levels of 273 µg/L in beers stored at 20 °C for 6 months [30]. In addition, the lower content of FURF in beers stored at 4 ± 1 °C (Figure 3) demonstrated that beer storage at low temperatures is a good strategy to retard the development of FURF and delay the appearance of bready and caramellic flavors during beer storage.

Strecker aldehydes (2-MP, 2-MB, and 3-MB) were detected in fresh beers below quantification limits (below 5.7 µg/L for 2-MP, below 3.9 µg/L for 2-MB, and below 4.2 µg/L for 3-MB). The concentration of 2-MP continuously increased during forced aging. The levels determined for long-term storage of 90 days represented an increase of about 270% (*p* < 0.05) up to the 14th day of storage. Malfliet et al. have described levels of 2-MP ranging from 9.5 to 38.2 µg/L in beers stored at 30 °C for 60 days [9], which are in accordance with the levels here reported for beers at comparable conditions (20 ± 1 µg/L at 63 days and 37 °C). Concerning natural and forced aging studies, no significant differences were found between the levels of 2-MP in beers stored at 20 ± 2 °C and in beers stored for 14 days at 37 ± 1 °C (*p* > 0.05). The levels of 2-MP found in both fresh and aged beers were below their flavor threshold (65 µg/L [14]).

The levels of 2-MB and 3-MB increased after 7 days of forced aging compared to fresh beers (Figure 3). However, only slight variations were observed for forced aged beers during the first 63 days (ranging between 3.9 ± 0.1 and 6.1 ± 0.1 µg/L for 2-MB and 4.5 ± 0.1 and 7.6 ± 0.3 µg/L for 3-MB). The maximum levels of 2-MB and 3-MB were achieved after 90 days of forced aging (15.2 ± 1.2 µg/L for 2-MB and 12.4 ± 0.7 µg/L for 3-MB), representing an increase of about 200% compared to forced aged beers, for 7 days. The determined contents of these Strecker aldehydes were in accordance with previous studies: ranging from 2.9 to 6.8 µg/L [8,9,31] for 2-MB and between 5.6 and 11.8 µg/L for 3-MB, in beers stored at 30 °C for 60 days [9]. As previously verified for the other aldehydes, storage at 4 ± 1 °C also delays the development of 2-MB and 3-MB. Moreover, natural beer aging during 3 and 6 months had no significant impact on the formation of 2-MB. On the other hand, a considerable variation in 3-MB levels was observed after 6 months at 20 ± 2 °C (4.7 ± 0.1 µg/L), similar to the levels found in beers stored for 7 days at 37 ± 1 °C. Nevertheless, the concentrations of 2-MB and 3-MB, observed in aged beers, were below their threshold values (46 µg/L^−1^ for 3-MB [14] and 35 µg/L for 2-MB [14]), which may indicate that no significant alterations on beer sensory quality, such as the presence of fruity, almond, chocolate, and malty notes, are expected.

ACET is mainly formed in beer by ethanol oxidation. As previously reported, nowadays, the most commercial beers are bottled under extremely low oxygen levels (<0.2 m/L), resulting in few oxidative aging reactions during storage [29]. Consequently, the development of ACET was compromised during aging, and the levels of this compound in fresh, natural, and forced aged beers, were similar. The rate, extent, and course of the Maillard reactions are influenced by several factors, including temperature and time. The Maillard products, especially FURF, are known as a key marker for the heat load on the mash, wort, and beer. Additionally, it is considered a marker for flavor staling in beer. The development of Maillard reaction products is favored by high temperatures (for example, 37 °C), and it can even be formed at a slow rate at lower temperatures [6]. This may explain the continuous development of FURF observed in forced aged beers during storage.

The Strecker degradation products are associated with the Maillard reactions since they are derived from the reaction between an α-dicarbonyl compound (mainly produced during the Maillard reaction) and an amino acid. In this work, three Strecker aldehydes were assessed derived from different amino acids present in beer, leucine, isoleucine, and valine, originating 3-MB, 2-MB, and 2-MP, respectively. The reported typical levels of valine (about 70 mg/L) are approximately 2-fold higher than the typical levels of leucine (about 40 mg/L^1^) and about 4-fold higher than isoleucine (about 20 mg/L) [32,33]. The disparity in the amount of 2-MP, 2-MB, and 3-MB amino precursors (valine, isoleucine, and leucine, respectively), may be the reason for the differences observed in the levels of these compounds during aging. 2-MP showed a constant increase during the forced aging, which may be related to the higher amounts of valine in beer. In contrast, the concentration of leucine and isoleucine in beer is lower and more time is needed for the production of 3-MB and 2-MB, respectively.

### 3.6. Chemometric Analysis

**Figure 4:**
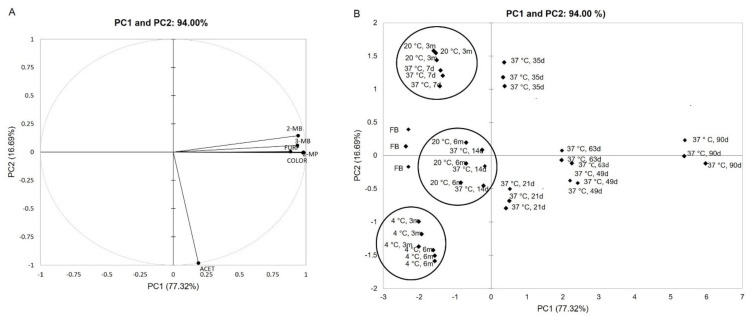
Principal component analysis (PCA) obtained (**A**) from the analyzed carbonyl compounds and beer color on PC1 and PC2 of all samples analyzed (fresh and natural and forced aged beers) and (**B**) 36 observations corresponding to each analyzed beer at different storage conditions (fresh beers (FB), 3 months (3 m) and 6 months (6 m) naturally aged at 20 ± 2 °C and at 4 ± 1 °C and forced aged beers (7–90 days at 37 ± 1 °C (7 d–90 d)), shown in the score plot.

Principal component analysis (PCA) was applied to reduce the redundant information in data and group the correlated responses into principal components. The PCA plot (1st principal component vs. 2nd principal component), exhibited in Figure 4, explains 94.00% of the total system variance. Each of the first and second principal components explains the variance of 77.32% (PC1) and 16.69% (PC2), respectively.

In the present study, PCA was applied for comparison of the studied beer samples (fresh beers (FB); beers maintained at 4 ± 1 °C for 3 and 6 months (3 m and 6 m); forced aged beers at 37 ± 1 °C between 7 and 90 days (7 d and 90 d); and naturally aged beers at 20 ± 2 °C for 3 and 6 months (3 m and 6 m)). In the loading plot (Figure 4A), measured parameters (aldehydes content and beer color) are displayed; in the score plot (Figure 4B) each point corresponds to a beer sample submitted to different storage conditions (different times and temperatures).

Analyzing the data in PC1 of Figure 4B, it is possible to observe that beers maintained at 4 ± 1 °C for 3 and 6 months have similar aging behavior and exhibit similar color and carbonyl profile; in comparison, beers maintained at 20 ± 2 °C for 3 months and at 37 ± 1 °C for 7 days, with similar color and carbonyl content, can be distinguished from beers stored at 4 ± 1 °C. Furthermore, beers stored at 20 ± 2 °C for 6 months and beers maintained at 37 ± 1 °C for 14 days can be grouped due to a similar score. Moreover, PC1 clearly differentiates fresh beers (on the negative side) from beers submitted to 37 ± 1 °C for 90 days (extreme forced aging conditions, in the positive side of PC1) (Figure 4B). These results allow us to confirm that beer color and the content of the evaluated staling aldehydes are key parameters to discriminate fresh beers from aged beers (Figure 4A,B).

## 4. Conclusions

In this work, a GDME method with simultaneous derivatization with HBA followed by HPLC-DAD analysis was developed and validated for the determination of beer staling aldehydes. The procedure was then applied to evaluate the impact of natural and forced aging on the levels of Strecker aldehydes, FURF, and ACET in beer. The optimized experimental extraction conditions were: headspace extraction for 20 min at 40 °C using HBA as a derivatizing agent. Recoveries between 96% and 114% were obtained for the studied aldehydes along with good intraday (RSD values between 1.0% to 3.4%) and interday (RSD values between 3.3% and 9.2%) precisions.

The validated method was then applied to evaluate the influence of beer storage on the releasing or development of aldehydes. Storing beer at low temperatures (4 ± 1 °C) greatly limited the formation of staling aldehydes in comparison to naturally and forced aged beers. The results herein reported also indicate that natural aging (20 ± 2 °C) during 3 and 6 months may mimic the aging induced by heat (37 ± 1 °C) for 7 and 14 days, respectively. Accordingly, temperature should be considered a critical parameter during beer storage and should be monitored in order to preserve beer sensory quality and organoleptic characteristics. The storage of beer at 4 ± 1 °C has been proved to be a good strategy to inhibit and delay the formation of staling aldehydes and their corresponding off-flavors.

## Figures and Tables

**Table 1:** Peak areas (mAU/min) of the assessed aldehydes (1 mg/L) in model solution, derivatized with DNPH and HBA using headspace extraction at the optimized conditions (30 and 20 min at 40 °C, respectively). Peak area means and the corresponding SD values are presented (*n* = 3). The different superscript letters represent differences considered statistically significant (*p* < 0.05) for the peak areas obtained for each compound using both derivatizing agents.

Compound	DNPH—Derivatives	HBA—Derivatives
	Peak Area ± SD	Peak Area ± SD
ACET	14.620 ± 1.168 ^a^	13.020 ± 0.707 ^a^
FURF	0.743 ± 0.080 ^a^	0.015 ± 0.001 ^b^
2-MB	10.822 ± 3.516 ^a^	2.115 ± 0.151 ^b^
3-MB	6.953 ± 2.856 ^a^	1.472 ± 0.055 ^b^
2-MP	12.192 ± 2.161 ^a^	2.565 ± 0.127 ^b^
IS	2.400 ± 0.214 ^a^	4.056 ± 0.259 ^b^

**Table 2:** Intraday and interday precisions obtained, considering the peak area (mAU/min) of each analyzed aldehyde in fresh beers spiked with standard aldehydes, using headspace and immersed extraction (*n* = 10).

	Headspace Extraction				Immersed Extraction			
	Precision				Precision			
	Intraday		Interday		Intraday		Interday	
Compound ^1^	Peak Area ± SD	RSD (%)	Peak Area ± SD	RSD (%)	Peak Area ± SD	RSD (%)	Peak Area ± SD	RSD (%)
ACET	75.48 ± 2.76	3.7	70.06 ± 6.83	9.7	45.33 ± 3.51	7.7	41.25 ± 6.40	15.5
FURF	0.45 ± 0.03	6.3	0.47 ± 0.04	9.2	0.78 ± 0.08	10.3	0.63 ± 0.13	20.6
2-MB	6.73 ± 0.24	3.6	6.81 ± 0.63	9.3	4.56 ± 0.21	4.6	3.90 ± 0.93	23.8
3-MB	11.43 ± 0.55	4.8	11.71 ± 0.99	8.5	6.78 ± 0.67	9.9	5.70 ± 1.50	26.3
2-MP	3.75 ± 0.10	2.7	3.77 ± 0.26	6.9	3.20 ± 0.28	8.8	2.74 ± 0.61	22.3
IS	14.58 ± 1.18	8.1	13.90 ± 1.32	9.5	11.05 ± 0.81	7.3	10.10 ± 1.35	13.4

^1^ACET (11.0 mg/L); FURF (2.0 mg/L); 2-MB (1.8 mg/L); 3-MB (1.8 mg/L); 2-MP (1.5 mg/L); IS (12.5 mg/L).

**Table 3:** Linear range, calibration curves, determination coefficients, limits of detection (LOD) and quantification (LOQ), precision and recovery of the proposed method.

Compound	Linear Range (µg/L)	Calibration Curve	r^2^	LOD (µg/L)	LOQ (µg/L)	Spike (µg/L)	Precision		Recovery (%)
							Intraday RSD (%)	Interday RSD (%)	
ACET	4000–30,000	y = (1.932 ± 0.080) x + (4404.907 ± 1095.240)	0.995	1857.7	6192.4	*	1.8	8.0	-
						High ^1^	2.4	3.4	103
FURF	100–1000	y = (0.161 ± 0.004) x + (−6.846 ± 1.788)	0.999	46.4	154.5	Low ^2^	3.3	5.1	97
						High ^1^	2.5	3.7	97
2-MP	3–40	y = (2.697 ± 0.056) x + (19.303 ±1.201)	0.999	1.7	5.7	Low ^2^	3.4	9.2	109
						High ^1^	1.0	8.2	114
2-MB	2–40	y = (4.200 ± 0.067) x + (10.944 ± 1.645)	0.999	1.2	3.9	Low ^2^	1.7	3.6	96
						High ^1^	1.6	3.7	97
3-MB	2–40	y = (5.324 ± 0.091) x + (11.385 ± 1.538)	0.999	1.3	4.2	Low ^2^	3.2	9.0	101
						High ^1^	1.2	3.3	102

* Beers were not spiked with a low level of ACET, considering its high levels in fresh beers. ^1^ High level: 11,101.3 µg/L for ACET; 960.8 µg/L for FURF; 36.1 µg/L for 2-MP and 34.4 µg/L for 2-MB and 3-MB. ^2^ Low level: 192.2 µg/L for FURF; 7.2 µg/L for 2-MP; 8.6 µg/L for 2-MB and 3-MB.

## Data Availability

The data presented in this study are available within the article.

## Sample Availability

Samples are not available from the authors.
